# Expression of MMP-14 and prognosis in digestive system carcinoma: a meta-analysis and databases validation

**DOI:** 10.7150/jca.36469

**Published:** 2020-01-01

**Authors:** Fujiao Duan, Zhen Peng, Jingjing Yin, Zhongyu Yang, Jia Shang

**Affiliations:** 1 Medical Research Office, Affiliated Cancer Hospital of Zhengzhou University, Zhengzhou, Henan, China.; 2 Department of Infectious Disease, Henan Provincial People's Hospital, People's Hospital of Zhengzhou University, Zhengzhou, Henan, 450003, China.; 3 College of Public Health, Zhengzhou University, Zhengzhou, Henan Province, China.; 4 College of Art and Science, The Ohio State University, Columbus, Ohio, US.

**Keywords:** MMP-14, expression, prognosis, digestive system carcinoma, meta-analysis.

## Abstract

**Background:** The Matrix metalloproteinase-14 (MMP-14) expression has been shown to be overexpressed in different cancers. However, there is no comprehensive quantitative evaluation of the MMP-14 prognostic value in digestive system carcinoma (DSC). The aim of this study is to explore the correlation between the MMP-14 expression and DSC prognosis**.**

**Methods:** We conducted a meta-analysis to estimate the association strength between MMP-14 expression and prognosis. GEPIA and Kaplan Meier plotters were used to assess overall survival (OS), disease-free survival (DFS)/progression-free survival (PFS) in DSC patients and the differential expression of MMP-14 in DSC tissues and adjacent tissues.

**Results:** A total of 20 studies including 2,519 patients with OS and 438 patients with DFS/PFS data were analyzed in evidence synthesis. Overall, the combined hazard ratio (HR) with 95% confidence interval (95% CI) was 1.98 (95%Cl: 1.77-2.22, *P*<0.001) for OS and 3.61 (95%Cl: 2.39-5.43, *P*<0.001) for DFS/PFS. For subgroup analyses, significant correlations were revealed between increased MMP-14 expression and poor OS in patients with gastric cancer (HR=2.21, 95%CI: 1.76-2.77, *P*<0.001), esophageal carcinoma (HR=2.01, 95%CI: 1.58-2.57, *P*<0.001), oral cancer (HR = 1.69, 95% CI: 1.30-2.20, *P* < 0.001) (HR=2.14, 95%CI 1.35-2.19, *P*<0.001) and hepatocarcinoma. In database verification analyses, the MMP-14 expression levels in normal tissues were significantly higher than that in DSC tissues, and significant associations were observed between high MMP-14 expression levels and poor prognosis.

**Conclusions:** The high expression levels of MMP-14 might predict poor prognosis in DSC. Larger prospective clinical cohort studies are required to validate the prognostic role.

## Introduction

As a non-communicable disease, cancer is recognized as the main cause of human death worldwide in the 21st century and the biggest obstacle to prolong life span; in which digestive system carcinoma (DSC) including esophageal carcinoma, gastric cancer, hepatocellular carcinoma, pancreatic cancer, gallbladder cancer, and colorectal cancer, etc., play undoubtedly important roles. Global cancer statistics showed that the incidence of colorectal cancer ranked third among all cancers, followed by gastric cancer (fifth), liver cancer (sixth) and esophageal cancer (seventh), and ranked second, third, fourth and sixth respectively in terms of mortality^1^. Although some biomarkers, for instance HER2 and alpha fetoprotein, have been applied to the diagnosis and prognosis of DSC, sometimes their clinical applications were greatly limited due to poor specificity and tumors heterogeneity 2, 3. Therefore, it is necessary to find novel biomarkers for more specific diagnostic and prognostic assessments.

Matrix metalloproteinases (MMPs) are essentially zinc-dependent endopeptidases existing in various tissues and more than 25 members are found currently in human beings 4, which are thought to play a key role in regulating metastasis and invasion and ultimately promoting tumor progression in a variety of cancers 5-7. MMPs have been thought to degrade a variety of extracellular matrix components previously^8^, ^9^, but studies in recent years have demonstrated that the function of MMPs can also involve in mediating extracellular, intercellular, and intracellular signaling pathways through complex interactions with multiple molecules^10^-^12^.

Recently, considerable interest has been focused on the important MMP family members. The predictive effects of MMP-1, MMP-9, MMP-12, MMP-14, and MMP-15 on adverse clinical outcomes of primary tumors have been identified by large microarray analyses 13, and MMP-9, MMP-11, and MMP-15 have also been shown to be associated with poor survival in tumors 14. None of these studies found any correlation between MMP and positive prognosis. Similarly, other studies reported the overexpression was closely linked to poor prognosis in nonsolid tumors 15, 16.

MMP-14 was the first characterized metalloproteinase in MMP family and naturally anchored to the cell membrane in an activated form. MMP-14, also known as the membrane-type MMP (MT1-MMP), have been shown to correlate with a variety of physiological functions and tumor-related behaviors such as migration 17, 18, invasion 17, 19, metastasis 17, 20, basement membrane remodeling 21, and angiogenesis 18, 20. Notably in the tumorigenesis and progression of various DSC, MMP-14 plays a considerably complicated role. For instance, studies have shown that down-regulation of MMP-14 could inhibit invasion, migration and angiogenesis in gastric carcinoma 22, 23. Researches by Li et al. have suggested that invasion and migration of hepatocellular carcinoma cells could be inhibited by targeting MMP-14^24^. Similar results can also be found in colorectal cancer 25, esophageal cancer 26, and pancreatic cancer 27. Nevertheless, the other argued that the role of MMP-14 in the formation and tumor development was controversial 28. It has been reported that MMP-14 could inhibit tumor angiogenesis by mediating the shedding of endoglin, thereby exerting a negative effect on tumor progression 29.

So far, there is not enough information on evidence-based medicine for prognostic significance of MMP-14 in DSC and the clinical application of targeting MMP-14 has also not been established yet. Therefore, we conducted this study to further clarify the part of MMP-14 plays particularly in the prognosis of DSC. Furthermore, we used GEPIA to provide differential expression between tumors and normal tissues and the evaluate the effect of MMP-14 expression on survival using 580 DSC patients with overall survival (OS) and disease free survival (DFS)/ progression-free survival (PFS) data.

## Material and Methods

This study was conducted based on the Meta-analysis of Observational Studies in Epidemiology (MOOSE) 30, Preferred Reporting Items for Systematic Reviews and Meta-Analysis (PRISMA) guidelines 31 and Population (patients with DSC), Intervention(Surgery), Comparator (expression levels), Outcomes (Survival) (PICO) methodology.

### Search strategy

We carried out a systematic literature search using PubMed, PMC, EMBASE, Cochrane Library, Web of Science, Wanfang (Chinese) and CNKI (Chine se) database through August 6, 2019. The combination terms for retrieval were “tumor” or “carcinoma” or “cancer” and “Membrane type-1 matrix metalloproteinase or MT1-MMP, Matrix metalloproteinase-14 or MMP-14, Matrix metalloproteinases or MMPs” and “outcome” or “prognosis” or “survival” (Supplementary Table 1). To further identify potential articles, we also manually retrieved bibliography of relevant studies that were not retrieved by databases exploration.

### Inclusion and exclusion criteria

Inclusive criteria: (1) expression levels of MMP-14 in DSC tissue was assessed by immunohistochemistry (IHC), (2) associations between MMP-14 expression levels in DSC and OS, DFS/PFS or other possible survival parameters were described, (3) the expression of MMP-14 were categorized into low and high groups, (4) hazard ratios (HR) with 95% confidence interval (95%CI) for survival analysis were presented or could be reckoned from the instance data.

Exclusive criteria: (1) reviews, case reports, letters, expert opinions and meta-analysis, (2) articles without usable data to calculate the HRs and corresponding 95% CIs, (3) neither English nor Chinese language, (4) duplicate publications.

If a study overlaps data from other published literature, we choose to publish the latest one and/or the largest sample size.

### Data extraction

The data of the following items were extracted from the eligible studies: Name of the first author, publication year, location of population, duration of follow-up, sample size, pathology subtypes, clinicopathological features, survival analyses results (univariate and/or multivariate analyses), HRs and *95% CI*s. Each study was considered as independent dataset. If *HRs* and *95% CIs* were not reported, they were extrapolated using the methods of Parmar 32 and Tierney 33.

### Methodological quality assessment

The methodological quality of eligible studies was evaluated by Newcastle-Ottawa Scale (NOS). NOS consists of three parts with a total of 9 points. Studies with NOS scores ≥ 6 points were considered as high-quality.

The specific Quality In Prognosis Studies (QUIPS) was assessed according to the method of Hayden et al 34. Estimates of potential bias include study participation, study attrition, prognostic factor measurement, outcome measurement, study confounding, statistical analysis, and reporting.

### MMP-14 expression profile and prognosis

GEPIA was used to evaluate the expression levels of MMP-14. GEPIA is an advanced interactive network server for analyzing the sequencing expression of RNA data of 9,736 cancers and 8,587 normal samples from the GTEx projects and TCGA, which are standard-based processing pipelines 35. It offers customizable features such as differentially expressed tumor/normal analysis, profiling according to pathological stages, types of cancer, survival analysis, related analysis, similar gene detection, and analysis of dimensionality reduction. Kaplan-Meier (KM) curve was applied to assess the effect of MMP-14 on survival using 580 DSC patients with OS and DFS/PFS data.

### Statistical analysis

The combined HRs with 95% CIs was conducted by Review Manager 5.3.5 (Cochrane Collaboration, Oxford, UK) to evaluate the relationship between MMP-14 expression levels and prognosis. Indicators of inter-study heterogeneity were tested by the Q-tests and *I*-squared (*I*^2^) 36. According to the results of heterogeneity analysis, when *P*-value of heterogeneity (*P*_heterogeneity_) ≥ 0.10 or *I*^2^ ≤ 50%, a fixed-effects model (Mantel-Haenszel method) 37 was applied to calculate the pooled effect size, otherwise (*P*_heterogeneity_ < 0.1 and *I*^2^ > 50%) the random-effects model (DerSimonian and Laird method)^38^ was employed, and the sources of heterogeneity was explored by meta-regression in STATA 13.1MP (StataCorp, College Station, TX, USA) 39.

For articles that didn't provide HRs with 95% CIs or *P*-value, Engauge Digitizer 10.0 (https://sourceforge.net/projects/digitizer/) was utilized to extract the original survival data from the KM curves. Subgroups analyses were conducted by evaluation methods of survival analysis, indicators of prognosis, and cancer subtypes (pathological type). Begg's 40 and Egger's test 41 were used to explore the publication bias in STATA 13.1MP.

If the 95% CI did not cover 1 and the pooled *HR* > 1, a statistical significance was considered. All *P*-values were two-sided and *P* < 0.05 was considered statistically significant. The KM plotter split is median, and the MMP-14 expression profile from DSC samples and paired normal tissues.

## Results

### Identification of the eligible studies

**Figure 1:**
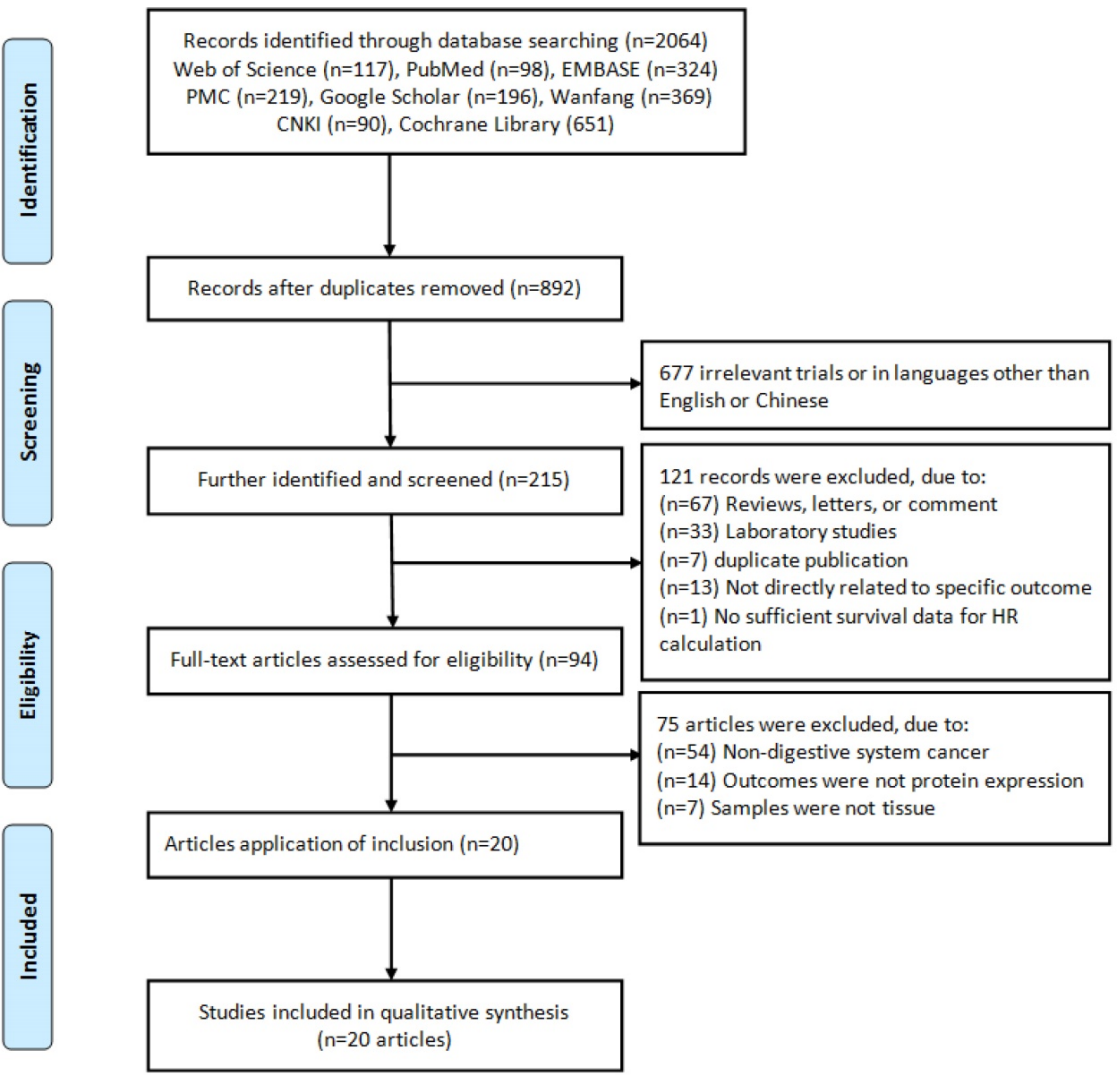
Flow chart of literature search and study selection.

A flow diagram of the literature search strategy was summarized in Figure 1, and a total of 2,064 records were retrieved from databases. By screening the titles and/or abstracts, we excluded 1,172 duplicates, 677 unrelated records or articles in languages other than English and Chinese, and 215 were further identified and screened, then retrieved 94 relevant full-text articles. 75 articles were further removed because of non-DSC or the samples were not detected in protein expression levels or were not derived from tissue. Finally, 20 eligible articles 42-61 were included in this meta-analysis (Table 1).

### Baseline characteristics of eligible studies

The basic characteristics of included studies were presented in Table 1. They were published from 2007 to 2019 and consist of 2,519 patients with OS and 438 patients with DFS/PFS from China and Japan. The study was based on the location of subjects to determine the country of study. The cancer types included gastric cancer, esophageal squamous cell carcinoma (ESCC), colorectal cancer (CRC), oral cancer, hepatocellular carcinoma (HCC), gallbladder carcinomas (GBC), and pancreatic cancer. The samples were all tissues and the methods of detection were IHC. The cut-off values of MMP-14 were immunohistochemistry score (HIS) and percentage of positive cells (PPC), most with HIS.

### Methodological quality assessment

**Figure 2:**
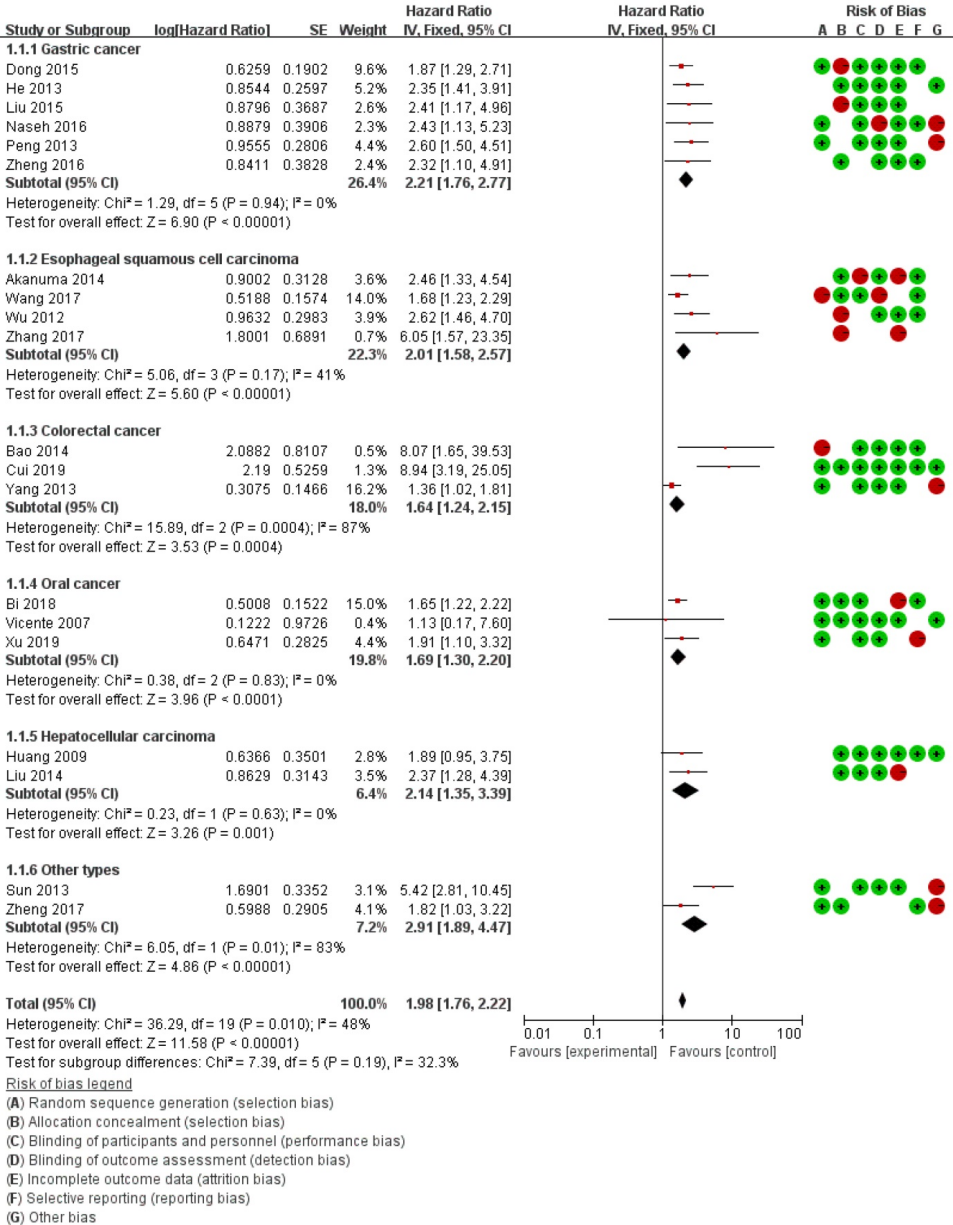
Forest plots for the relationship between MMP-14 expression and overall survival.

According to QUIPS, the quality assessment for eligible studies was summaried in Table 2. The risk of bias domains legend was presented in Figure 2. According to NOS (Supplementary Table 2), yielded scores ranging from 5 to 9, with a mean score of 7.05, 90.00% (18/20) of these studies were considered as high-quality (quality score≥6).

### Expression in DSC tissue

The heterogeneity of MMP-14 for OS was at the critical value of the statistics (*P*_heterogeneity_ = 0.01 and *I*^2^ = 48%). According to the preconditions, the fixed effect was applied to calculate the combined HRs for MMP-14. Overall, the pooled analysis of the 17 studies indicated that MMP-14 expression was significantly associated with OS (HR = 1.98, 95% Cl: 1.76-2.22, *P* < 0.001) and DFS/PFS (HR = 3.61, 95% Cl: 2.39-5.43, *P* < 0.001) (Table 3).

Subgroup analysis was carried out based on statistical approach, and the results revealed that high MMP-14 expression in both log rank (HR = 1.86, 95% CI: 1.57-2.28, *P* < 0.001) and multivariate analysis (HR = 2.64, 95% CI: 1.95-3.58, *P* < 0.001) were significant associated with poor OS (Table 3). Meanwhile, stratified analysis based on cut-off value showed the high expression of MMP-14 for IHS (HR = 2.18, 95% CI: 1.69-2.81, *P* < 0.001) and PPC (HR = 2.48, 95% CI: 1.92-3.20, *P* < 0.001) were statistically significant with the poor OS, respectively.

According to cancer subtype, we conducted subgroup analysis of gastric cancer, ESCC, colorectal cancer, HCC and other types, respectively. It revealed a significant correlation between increased MMP-14 and poor OS in gastric cancer patients (HR = 2.21, 95% CI: 1.76-2.77, *P* < 0.001), ESCC (HR = 2.01, 95% CI: 1.58-2.57, *P* < 0.001), HCC (HR = 2.14, 95% CI 1.35-2.19, *P* < 0.001), oral cancer (HR = 1.69, 95% CI: 1.30-3.20, *P* < 0.001) and other types of DSC (HR = 3.10, 95% CI 1.06-9.03, *P* < 0.001). For subgroups analyses, there was no heterogeneity among subgroups (𝐼^2^ = 0%, 𝑃 = 0.90) (Table 3).

### Test of heterogeneity

Nineteen OS related datasets displayed the critical value of the statistics heterogeneity (*P*_heterogeneity_ = 0.01 and *I*^2^ = 48%). To ensure the robustness and statistical effectiveness of the results, meta-regression was employed to investigated sources of heterogeneity for OS, including year of publication, cancer types, ethnicity (Asians or Caucasians), language (English or Chinese), sample size (100 as the boundary), cut-off and follow-up. The result revealed that there was no altered by above characteristics (Table 4).

### Sensitivity analysis and publication bias

To verify the robustness of our results, sensitivity analysis was performed by removing one study at a time and recalculating pooled HR. The results did not substantially alter the combined HR, indicating that this meta-analysis was reliable. (Supplementary Figure 1).

The funnel plots shape was basically symmetrical (Supplementary Figure 2), and Begg's and Egger's tests indicated that there is no substantial publication bias (Supplementary Table 3).

### MMP-14 expression profile and prognosis

**Figure 3:**
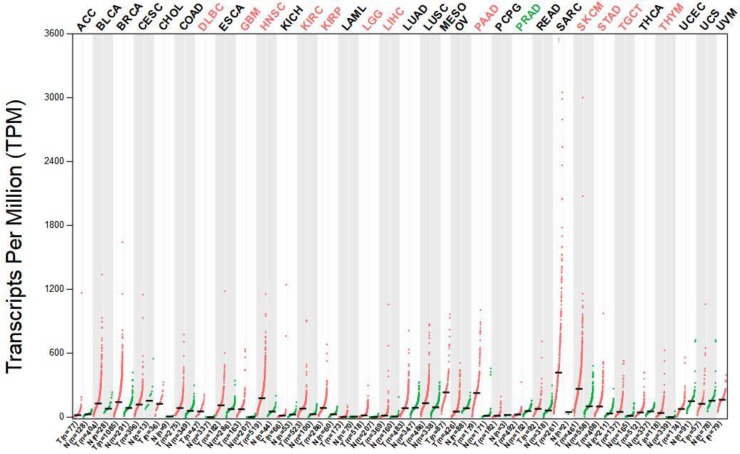
The expression of MMP-14 in DSC tissues and adjacent normal tissues.

The median expression of tumor (red dots) and normal (green dots) samples were presented in Figure 3. Each dot represents a sample expression level, Gene expression profiles consist of various tumor tissues and paired normal tissues. The results indicated that the MMP-14 expression level in DSC tissues was significantly higher than that in normal tissues (Supplementary Table 4).

**Figure 4:**
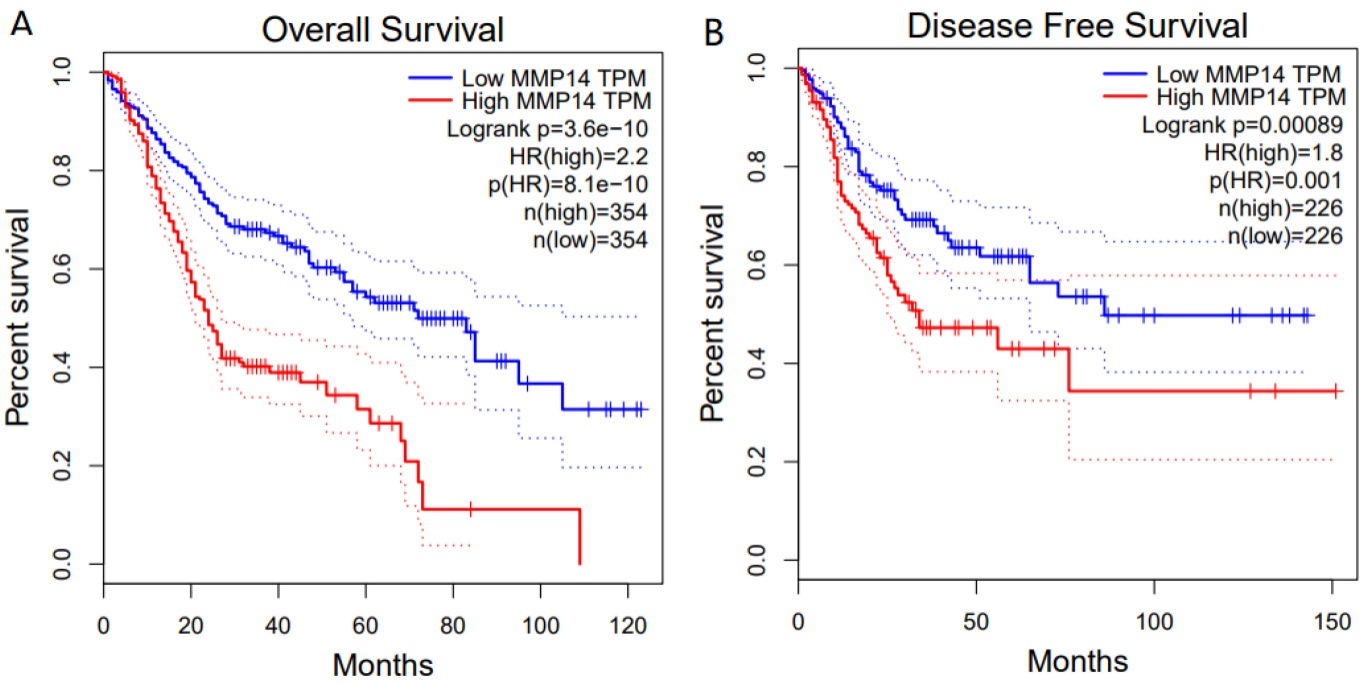
Kaplan-Meier survival curves for OS and DFS according to MMP-14 expression in patients with DSC. OS (A) and DFS (B) of patients with high vs. low MMP-14 expression are shown.

For OS, a highly significant correlation was revealed between high MMP-14 expression level and poor prognosis (HR = 2.2, *P*<0.001) (Figure 4A). Similarly, MMP-14 expression was significantly associated with DFS (HR = 1.8, *P* < 0.001) (Figure 4B).

## Discussion

Over the last few years, overexpression of MMP-14 has been proved to be an independent prognostic factor for several cancers, its prognostic significance is adverse 62. However, the clinical prognostic significance of MMP-14 is still not characterized in DSC. Thus, this meta-analysis was performed to provide more accurate evidence for the prognostic value of MMP-14 in patients with DSC. Then, the expression data of cancer and paired normal tissues from GTEx projects and TCGA was used to illustrate our result.

The accumulated evidence indicates that members of the MMP protein family are related to the progression and tumorigenesis including metastasis and invasion of various cancers, and ultimately affect patient survival. MMP-14 was the first membrane type MMP discovered, it is a member of membrane-type MMP and participates in many biological processes due to its ability to promote angiogenesis and matrix degradation 63. Changing the MMP-14 level may leads to the rearrangement of cytoskeleton and invasion and migration 28.

In this study, we only included the studies that examined the MMP-14 expression in tissues (tumor and paired normal tissues) by immunohistochemistry to ensure the reliability and consistency our results. We found that high expression of MMP-14 may be an independent poor prognostic factor (OS, HR = 1.98, 95% Cl: 1.76-2.22, *P* < 0.001 and DFS/PFS, HR = 3.61, 95% Cl: 2.39-5.43, *P* < 0.001), particularly in multivariate analysis group (HR = 2.64, 95% CI: 1.95-3.58, *P* < 0.001), and patients with gastric cancer (HR = 2.21, 95% CI: 1.76-2.77, *P* < 0.001) and HCC (HR = 2.14, 95% CI: 1.35-2.19, *P* < 0.001) and oral cancer(HR = 1.69, 95% CI: 1.30-3.20, *P* < 0.001). Our findings of gastric cancer were consistent with another similar study 64, nevertheless, no correlation was observed between the MMP-14 expression and the prognosis of colorectal cancer.

In order to be able to infer reliable results, we first tested the prognostic significance of MMP14 in the GTEx projects and TCGA, and then verified our results in these two databases. In the GTEx projects, the MMP-14 expression in DSC tissues was significantly higher than that in normal tissues, Moreover, we used the 580 paired DSC and normal tissues from the TCGA database with OS and DFS data, the MMP14 expression in DSC was significantly higher than that in normal control group. All these findings are further confirmed our conclusion, and indicated that MMP-14 may be an independent prognostic factor for both OS and DFS/PFS in patients with DSC.

MMP-14 promotes angiogenesis by promoting the expression of vascular epidermal growth factor and releasing biologically active extracellular matrix products 65. MMP-14 is overexpressed in most human cancer, and the published studies have demonstrated that overexpression of MMP-14 in various cancers is associated with poor prognosis 66. Our findings of cancer subtype analysis were consistent with this study.

MMP-14 has been showed to be activated by TGFβ1, and then plays a complex role in tumor formation, angiogenesis and invasion through destruction and reconstruction of the basement membrane 67.TGFβ1 signaling promotes migration, progression and growth of the late carcinomas. Amara et al. found that stimulation of soluble factors secreted by cancer cells activates MMP-14. One of the effectors is TGFβ1, which differentiates fibroblasts in the matrix and stimulates MMP-14 expression and activation 68. The published studies of MMP14 focused on angiogenesis and tumor invasion 69, 70. Previous studies have shown MMP-14 was highly expressed in different tumor tissues, and its expression could promote the migration, invasion and metastasis of tumor cells *in vitro* and *in vivo* 71. Potentially, MMP14 may play a crucial role in different biological processes of cancer and normal tissues 72.

Although our main results are robust, there are some limitations. Firstly, not all eligible studies provide multivariate adjusted HRs, so, a portion of HRs with the 95% CIs were extracted from survival curves. Although we have subgroup analysis based on statistical approach, these calculated might be generated several tiny errors. Secondly, the algorithms of cut-off values of MMP-14 expression were different, which may lead to deviation of the true values. Thirdly, although there was no statistical evidence of publication bias, most eligible studies are in China, which may lead to publication bias. Finally, bioinformatics analyses need to be validated by more experimental and biological study. Despite these limitations, this study about association between MMP-14 and DSC prognosis was certainly warranted.

In conclusion, our study provided the first evidence of MMP-14 in the prognosis of DSC, which strongly suggested that MMP-14 has potential in prediction of treatment outcomes and may be a potential independent prognostic factor in DSC. Furthermore, high-quality and multicenter clinical studies should be carried out to further clarify the significance of MMP-14 in survival outcomes of DSC.

## Supplementary Material

Supplementary figures and tables.

## Figures and Tables

**Table 1:** Clinicopathological characteristics of eligible studies.

Author	Year	Country	Ethnicity	Number		Histology	Tumor stage	Follow-up (Months)	Cut-off	Analysis	Outcome
				OS	DFS/PFS						
Cui et al 42	2019	China	Asian	218	DFS,218	Colorectal cancer	TNM (NA)	60	IHS≥4	Cox regression	HR/SC
Xu et al 43	2019	China	Asian	80	PFS,80	Oral cancer	TNM, I-IV	120	IHS≥4	Kaplan-Meier	SC
Bi et al 44	2018	China	Asian	83		Oral cancer	TNM, I-IV	100	IHS≥3	Kaplan-Meier	SC
Wang et al 45	2017	China	Asian	204		ESCC	TNM, I-Ⅲ	120	IHS≥6	Kaplan-Meier	SC
Zhang et al 46	2017	China	Asian	142		ESCC	TNM(NA)	84	HIS>4	Cox regression	HR/SC
Zheng et al 47	2017	China	Asian	68		Pancreatic cancer	TNM, I-IV	50	IHS≥4	Kaplan-Meier	SC
Naseh et al 48	2016	Iran	Caucasian	96		Gastric Cancer	TNM I-IV	150	IHS≥4	Cox regression	HR/SC
Zheng et al 49	2016	China	Asian	50		Gastric cancer	TNM I-IV	60	PPC≥25%	Cox regression	HR/SC
Dong et al 50	2015	China	Asian	205		Gastric cancer	TNM I-IV	100	IHS≥5	Cox regression	HR/SC
Liu et al 51	2015	China	Asian	95		Gastric Cancer	NA	60	PPC≥25%	Kaplan-Meier	SC
Akanuma et al 52	2014	Japan	Asian	140	DFS,140	ESCC	TNM, I-IV	120	PPC≥25%	Cox regression	HR/SC
Bao et al 53	2014	China	Asian	90		Colorectal cancer	Dukes'stage	81	IHS≥3	Cox regression	HR
Liu et al 54	2014	China	Asian	102		HCC	NA	60	PPC≥20%	Kaplan-Meier	SC
He et al 55	2013	China	Asian	205		Gastric Cancer	TNM I-IV	100	IHS>4	Cox regression	HR/SC
Peng et al 56	2013	China	Asian	184		Gastric cancer	TNM I-IV	108	PPC≥25%	Cox regression	HR/SC
Sun et al 57	2013	China	Asian	89		GBC	NSS, S1-S5	60	IHS(NA)	Cox regression	HR/SC
Yang et al 58	2013	China	Asian	243		Colorectal cancer	Dukes'stage	108	IHS>4	Cox regression	HR/SC
Wu et al 59	2012	China	Asian	95		ESCC	NA	60	PPC≥20%	Kaplan-Meier	SC
Huang et al 60	2009	China	Asian	61		HCC	NA	60	IHS≥2	Cox regression	HR/SC
Vicente et al^61^	2007	Spain	Caucasian	69		Oral cancer	TNM, I-IV	132	IHS≥3	Kaplan-Meier	SC

HCC, hepatocellular carcinoma; ESCC, esophageal squamous cell carcinoma; GBC, gallbladder carcinomas; DFS, disease free survival; HR, Hazard ratio; SC, survival curve; IHS, immunohistochemistry score; NSS, Nevin stage system; PPC, Percentage of positive cells.

**Table 2:** Quality assessment of included studies based on the Quality In Prognosis Studies (QUIPS).

Study	Quality evaluation of prognosis study						Total Score^a^	Level of Evidence^b^
	Study Participation	Study Attrition	Prognostic Factor Measurement	Outcome Measurement	Study Confounding	Statistical Analysis and Reporting		
Cui et al 42	Yes	Partly	Yes	Yes	Partly	Yes	9	1b
Xu et al 43	Yes	Partly	Yes	Partly	Partly	Partly	6	2b
Bi et al 44	Yes	Partly	Yes	Partly	Partly	Partly	8	2b
Wang et al 45	Partly	Partly	Yes	Yes	Partly	Partly	7	2b
Zhang et al 46	Yes	Partly	Yes	Yes	Partly	Yes	8	2b
Zheng et al 47	Partly	Partly	Yes	Yes	Partly	Partly	7	2b
Naseh et al 48	Partly	Partly	Yes	Yes	Partly	Yes	5	2b
Zheng et al 49	Partly	Partly	Yes	Yes	Partly	Yes	7	2b
Dong et al 50	Partly	Partly	Yes	Yes	Partly	Yes	7	2b
Liu et al 51	Partly	Partly	Yes	Partly	Partly	Partly	7	2b
Akanuma et al 52	Partly	Partly	Yes	Partly	Partly	Partly	5	2b
Bao et al 53	Partly	Partly	Yes	Yes	Partly	Partly	7	2b
Liu et al 54	Partly	Partly	Yes	Partly	Partly	Partly	7	2b
He et al 55	Partly	Partly	Yes	Yes	Partly	Yes	9	2b
Peng et al 56	Yes	Partly	Yes	Yes	Partly	Yes	6	2b
Sun et al 57	Yes	Partly	Yes	Yes	Partly	Yes	6	2b
Yang et al 58	Yes	Partly	Yes	Yes	Partly	Yes	6	2b
Wu et al 59	Partly	Partly	Yes	Partly	Partly	Partly	7	2b
Huang et al 60	Partly	Partly	Yes	Yes	Partly	Yes	8	2b
Vicente et al^61^	Yes	Partly	Yes	Yes	Partly	Partly	9	2b

^a^ Quality assessment of included studies based on the Newcastle-Ottawa Scale.^b^ The levels of evidence were estimated for all included studies with the Oxford Centre for Evidence Based Medicine criteria.

**Table 3:** Main results of pooled HRs in the meta-analysis.

Comparisons	Heterogeneity test			Summary HR (95% CI)	Hypothesis test		Model	Studies
	*Q*	*P*	*I^2^*(%)		*Z*	*P*		
Total (High vs. Low)								
OS	36.29	0.01	48	1.98(1.76,2.22)	11.58	<0.001	Fixed	20
DFS/PFS	2.38	0.30	16	3.61(2.39,5.43)	6.14	<0.001	Fixed	3
OS								
*Analysis*								
Log rank (KM)	4.05	0.77	0	1.86(1.57,2.28)	7.17	<0.001	Fixed	8
Multivariate analysis (Cox)	31.16	0.001	65	2.64(1.95,3.58)	6.26	<0.001	Random	12
*Cut-off*								
IHS	31.61	0.002	62	2.18(1.69,2.81)	6.02	<0.001	Random	13
PPC	0.12	0.99	0	2.48(1.92,3.20)	6.95	<0.001	Fixed	6
*Cancer subtypes*								
Gastric Cancer	1.29	0.94	0	2.21(1.76,2.77)	6.90	<0.001	Fixed	6
ESCC	5.06	0.17	41	2.01(1.58,2.57)	5.60	<0.001	Fixed	4
Colorectal cancer	15.89	0.00	87	4.16(0.93,18.56)	1.87	0.06	Random	3
Oral cancer	0.38	0.83	0	1.69(1.30,2.20)	3.96	<0.001	Fixed	3
HCC	0.23	0.63	0	2.14(1.35,2.19)	3.26	<0.001	Fixed	2
Other types	6.05	0.01	83	3.10(1.06,9.03)	2.07	0.04	Random	2
Test for subgroup difference	1.06	0.90	0					

KM, survival data from a Kaplan-Meier curve; Cox, survival data from a multivariate Cox regression analysis; Other types of cancer include Pancreatic cancer and gallbladder carcinomas; IHS, immunohistochemistry score; PPC, Percentage of positive cells.

**Table 4:** The results of heterogeneity test.

Comparisons	Coef.	Std. Err.	*t*	*P*	95% CI
Publication year	0.172	0.315	0.55	0.593	-0.751-0.408
Cancer types	0.020	0.094	0.21	0.836	-0.185-0.214
Language	-0.172	0.266	-0.65	0.531	-0.751-0.408
Ethnic	0.205	0.562	-0.37	0.721	-1.428-1.018
Cut-off	0.007	0.328	0.02	0.983	-0.707-0.721
Follow up	-0.577	0.605	-0.95	0.360	-1.990-0.742
Sample size	-0.383	0.293	1.31	0.215	-0.254-1.020

## References

[1] Bray F, Ferlay J, Soerjomataram I, Siegel RL, Torre LA, Jemal A (2018). Global cancer statistics 2018: GLOBOCAN estimates of incidence and mortality worldwide for 36 cancers in 185 countries. CA: A Cancer Journal for Clinicians.

[2] Grabsch H, Sivakumar S, Gray S, Gabbert HE, Muller W (2010). HER2 expression in gastric cancer: Rare, heterogeneous and of no prognostic value - conclusions from 924 cases of two independent series. Cellular oncology: the official journal of the International Society for Cellular Oncology.

[3] Fu J, Wang H (2018). Precision diagnosis and treatment of liver cancer in China. Cancer letters.

[4] Nagase H, Visse R, Murphy G (2006). Structure and function of matrix metalloproteinases and TIMPs. Cardiovascular research.

[5] Rowe RG, Weiss SJ (2009). Navigating ECM barriers at the invasive front: the cancer cell-stroma interface. Annual review of cell and developmental biology.

[6] Overall CM, Kleifeld O (2006). Tumour microenvironment - opinion: validating matrix metalloproteinases as drug targets and anti-targets for cancer therapy. Nature reviews Cancer.

[7] Conlon GA, Murray GI (2018). Recent advances in understanding the roles of matrix metalloproteinases in tumour invasion and metastasis. The Journal of pathology.

[8] Vandooren J, Van den Steen PE, Opdenakker G (2013). Biochemistry and molecular biology of gelatinase B or matrix metalloproteinase-9 (MMP-9): the next decade. Critical reviews in biochemistry and molecular biology.

[9] Bonnans C, Chou J, Werb Z (2014). Remodelling the extracellular matrix in development and disease. Nature reviews Molecular cell biology.

[10] Butler GS, Overall CM (2009). Updated biological roles for matrix metalloproteinases and new "intracellular" substrates revealed by degradomics. Biochemistry.

[11] Cauwe B, Opdenakker G (2010). Intracellular substrate cleavage: a novel dimension in the biochemistry, biology and pathology of matrix metalloproteinases. Critical reviews in biochemistry and molecular biology.

[12] Sternlicht MD, Werb Z (2001). How matrix metalloproteinases regulate cell behavior. Annual review of cell and developmental biology.

[13] McGowan PM, Duffy MJ (2008). Matrix metalloproteinase expression and outcome in patients with breast cancer: analysis of a published database. Annals of oncology: official journal of the European Society for Medical Oncology / ESMO.

[14] Christina C, Shah SP, Suet-Feung C, Gulisa T, Rueda OM, Dunning MJ (2012). The genomic and transcriptomic architecture of 2,000 breast tumours reveals novel subgroups. Nature.

[15] Lin LI, Lin DT, Chang CJ, Lee CY, Tang JL, Tien HF (2002). Marrow matrix metalloproteinases (MMPs) and tissue inhibitors of MMP in acute leukaemia: potential role of MMP-9 as a surrogate marker to monitor leukaemic status in patients with acute myelogenous leukaemia. British Journal of Haematology.

[16] Yu XF, Han ZC (2006). Matrix metalloproteinases in bone marrow: roles of gelatinases in physiological hematopoiesis and hematopoietic malignancies. Histology & Histopathology.

[17] Remacle AG, Cieplak P, Nam DH, Shiryaev SA, Ge X, Strongin AY (2017). Selective function-blocking monoclonal human antibody highlights the important role of membrane type-1 matrix metalloproteinase (MT1-MMP) in metastasis. Oncotarget.

[18] Li Y, Kuscu C, Banach A, Zhang Q, Pulkoski-Gross A, Kim D (2015). miR-181a-5p Inhibits Cancer Cell Migration and Angiogenesis via Downregulation of Matrix Metalloproteinase-14. Cancer research.

[19] Seiki M (2003). Membrane-type 1 matrix metalloproteinase: a key enzyme for tumor invasion. Cancer letters.

[20] Ling B, Watt K, Banerjee S, Newsted D, Truesdell P, Adams J (2017). A novel immunotherapy targeting MMP-14 limits hypoxia, immune suppression and metastasis in triple-negative breast cancer models. Oncotarget.

[21] Itoh Y, Seiki M (2006). MT1-MMP: a potent modifier of pericellular microenvironment. Journal of cellular physiology.

[22] Zheng L, Chen Y, Ye L, Jiao W, Song H, Mei H (2017). miRNA-584-3p inhibits gastric cancer progression by repressing Yin Yang 1- facilitated MMP-14 expression. Scientific reports.

[23] Zheng L, Li D, Xiang X, Tong L, Qi M, Pu J (2013). Methyl jasmonate abolishes the migration, invasion and angiogenesis of gastric cancer cells through down-regulation of matrix metalloproteinase 14. BMC cancer.

[24] Li T, Xie J, Shen C, Cheng D, Shi Y, Wu Z (2014). miR-150-5p inhibits hepatoma cell migration and invasion by targeting MMP14. PloS one.

[25] Yang B, Gao J, Rao Z, Shen Q (2013). Clinicopathological and prognostic significance of alpha5beta1-integrin and MMP-14 expressions in colorectal cancer. Neoplasma.

[26] Pang L, Li Q, Li S, He J, Cao W, Lan J (2016). Membrane type 1-matrix metalloproteinase induces epithelial-to-mesenchymal transition in esophageal squamous cell carcinoma: Observations from clinical and *in vitro* analyses. Scientific reports.

[27] Knapinska AM, Estrada CA, Fields GB (2017). The Roles of Matrix Metalloproteinases in Pancreatic Cancer. Progress in molecular biology and translational science.

[28] Pietraszek-Gremplewicz K, Karamanou K, Niang A, Dauchez M, Belloy N, Maquart FX (2019). Small leucine-rich proteoglycans and matrix metalloproteinase-14: Key partners?. Matrix biology: journal of the International Society for Matrix Biology.

[29] Hawinkels LJ, Kuiper P, Wiercinska E, Verspaget HW, Liu Z, Pardali E (2010). Matrix metalloproteinase-14 (MT1-MMP)-mediated endoglin shedding inhibits tumor angiogenesis. Cancer research.

[30] Stroup DF, Berlin JA, Morton SC, Olkin I, Williamson GD, Rennie D (2000). Meta-analysis of observational studies in epidemiology: a proposal for reporting. Meta-analysis Of Observational Studies in Epidemiology (MOOSE) group. JAMA.

[31] Moher D, Liberati A, Tetzlaff J, Altman DG (2009). Preferred reporting items for systematic reviews and meta-analyses: the PRISMA statement. Annals of internal medicine.

[32] Parmar MK, Torri V, Stewart L (1998). Extracting summary statistics to perform meta-analyses of the published literature for survival endpoints. Stat Med.

[33] Tierney JF, Stewart LA, Ghersi D, Burdett S, Sydes MR (2007). Practical methods for incorporating summary time-to-event data into meta-analysis. Trials.

[34] Hayden JA, Windt DA, Van Der, Cartwright JL, Pierre CT, Claire B (2013). Assessing bias in studies of prognostic factors. Annals of internal medicine.

[35] Tang Z, Li C, Kang B, Gao G, Li C, Zhang Z (2017). GEPIA: a web server for cancer and normal gene expression profiling and interactive analyses. Nucleic acids research.

[36] Higgins JP, Thompson SG, Deeks JJ, Altman DG (2003). Measuring inconsistency in meta-analyses. BMJ.

[37] Mantel N, Haenszel W (1959). Statistical aspects of the analysis of data from retrospective studies of disease. Journal of the National Cancer Institute.

[38] Dersimonian R, Laird N (1986). Meta-analysis in clinical trials. Controlled clinical trials.

[39] Thompson SG, Higgins JP (2002). How should meta-regression analyses be undertaken and interpreted?. Stat Med.

[40] Begg CB, Mazumdar M (1994). Operating characteristics of a rank correlation test for publication bias. Biometrics.

[41] Egger M, Davey Smith G, Schneider M, Minder C (1997). Bias in meta-analysis detected by a simple, graphical test. BMJ.

[42] Cui G, Feng C, Zhanwei D, Ling G (2019). MMP14 predicts a poor prognosis in patients with colorectal cancer. Human Pathology.

[43] Xu YT, Liu YH (2019). Expression levels and clinical significances of MMP-14 and HIF-1α in oral squamous cell carcinoma. Biomedical Engineering and Clinical Medicine.

[44] Bi Y, Zhou T, Jia YH, Xu ZN, Zhao XY, Zhang ZB (2018). A clinicopathological study of the prognosis and metastasis of MMP-14 in oral cancer patients. Stomatology.

[45] Wang C, Li Z, Shao F, Yang X, Feng X, Shi S (2017). High expression of Collagen Triple Helix Repeat Containing 1 (CTHRC1) facilitates progression of oesophageal squamous cell carcinoma through MAPK/MEK/ERK/FRA-1 activation. Journal of Experimental & Clinical Cancer Research Cr.

[46] Zhang Q The clinical significant of YAP1, MMP14 and AJUBA in ESCC and the prognostic role of YAP1 in common alimentary system cancers: a Meta-analysis: Hebei Medical University; 2017[Chinese Thesis].

[47] Zheng L Preliminary study of collagen binding peptide CBP1495 hepatic fibrosis imaging and MMP-14 activated cyclic CBP1495 in pancreatic cancer: Third Military Medical University; 2017[Chinese Thesis].

[48] Naseh G, Mohammadifard M, Mohammadifard M (2016). Upregulation of cyclin-dependent kinase 7 and matrix metalloproteinase-14 expression contribute to metastatic properties of gastric cancer. Iubmb Life.

[49] Zheng L, Jiao W, Mei H, Song H, Li D, Xiang X (2016). miRNA-337-3p inhibits gastric cancer progression through repressing myeloid zinc finger 1-facilitated expression of matrix metalloproteinase 14. Oncotarget.

[50] Yichen D, Guohua C, Mingming G, Xia T (2015). Increased expression of MMP14 correlates with the poor prognosis of Chinese patients with gastric cancer. Gene.

[51] Liu A, Liu Y, Zhang H, Wang X (2015). Expression and correlation of SOX-18, MMP-2 and MMP-14 in gastric adenocarcinoma.

[52] Akanuma N, Hoshino I, Akutsu Y, Murakami K, Isozaki Y, Maruyama T (2014). MicroRNA-133a regulates the mRNAs of two invadopodia-related proteins, FSCN1 and MMP14, in esophageal cancer. British Journal of Cancer.

[53] Bao B, Xu LI, Xiao M, Gastroenterology DO, Hospital FA, University S (2014). Expression and clinical siginificance of MMP-14 in colorectal carcinoma. Jiangsu Medical Journal. 2014.

[54] Min L, Xia Z, Hou E, Wang S (2014). Expressions and clinical significance of Glypican3,MMP-9 and MMP-14 in primary hepatocellular carcinoma. Chongqing Medicine. 2014.

[55] Liang H, Dake C, Xia L, Jianyong Z, Shanhong L, Jipeng L (2013). Matrix metalloproteinase-14 is a negative prognostic marker for patients with gastric cancer. Dig Dis Sci.

[56] Peng CW (2013). Combined features based on MT1-MMP expression, CD11b + immunocytes density and LNR predict clinical outcomes of gastric cancer. Journal of Translational Medicine.

[57] Sun W, Fan YZ, Zhang WZ (2013). MMP-2 and MT1-MMP contribute to vasculogenic mimicry and poor prognosis in human primary gallbladder carcinomas. Surgical Research & New Technique.

[58] Yang B, Gao J, Rao Z, Shen Q (2013). Clinicopathological and prognostic significance of α5β1-integrin and MMP-14 expressions in colorectal cancer. Neoplasma.

[59] Wu X, Wu G, Peng C, Zhou Y, Wu Y, Hu D (2012). Expression of matrix metalloproteinase-14 and cathepsin D in esophageal squamous cell carcinoma and their prognostic value. Chinese Journal of Clinical Pharmacology and Therapeutics.

[60] Huang S Expression and significance of RECK and MMP-14 protein in human hepatocellular carcinoma: Guangxi Medical University; 2009[Chinese Thesis].

[61] de Vicente JC, Lequerica-Fernandez P, Santamaria J, Fresno MF (2007). Expression of MMP-7 and MT1-MMP in oral squamous cell carcinoma as predictive indicator for tumor invasion and prognosis. Journal of oral pathology & medicine: official publication of the International Association of Oral Pathologists and the American Academy of Oral Pathology.

[62] Tu C, Zhou J, Yuan L (2016). Letter regarding "MT1-MMP is not a good prognosticator of cancer survival: evidence from 11 studies" by Wu KP et al. Tumour biology: the journal of the International Society for Oncodevelopmental Biology and Medicine.

[63] Cepeda MA, Evered CL, Pelling JJH, Damjanovski S (2016). Inhibition of MT1-MMP proteolytic function and ERK1/2 signalling influences cell migration and invasion through changes in MMP-2 and MMP-9 levels. Journal of Cell Communication & Signaling.

[64] Wu KP, Li Q, Lin FX, Li J, Wu LM, Li W (2014). MT1-MMP is not a good prognosticator of cancer survival: evidence from 11 studies. Tumour Biology the Journal of the International Society for Oncodevelopmental Biology & Medicine.

[65] Yuichiro M, Mayumi O, Akihiko K, Toshiro Y, Yuji B, Masayoshi K (2006). Tumor growth suppression in pancreatic cancer by a putative metastasis suppressor gene Cap43/NDRG1/Drg-1 through modulation of angiogenesis. Cancer research.

[66] Ferlay J, Soerjomataram I, Dikshit R, Eser S, Mathers C, Rebelo M (2015). Cancer incidence and mortality worldwide: sources, methods and major patterns in GLOBOCAN 2012. International journal of cancer Journal international du cancer.

[67] Juha H, Lotta S, Nami S, Kaisa L, Jorma KO (2010). Secretion of active membrane type 1 matrix metalloproteinase (MMP-14) into extracellular space in microvesicular exosomes. Journal of Cellular Biochemistry.

[68] Neri A, Martina T, Matthew B (2018). Chemical tools for selective activity profiling of endogenously expressed MMP-14 in multicellular models. ACS Chemical Biology.

[69] Hui P, Xu X, Xu L, Hui G, Wu S, Lan Q (2015). Expression of MMP14 in invasive pituitary adenomas: relationship to invasion and angiogenesis. International Journal of Clinical & Experimental Pathology.

[70] Zhou M, Zhang XY, Yu X (2017). Overexpression of the long non-coding RNA SPRY4-IT1 promotes tumor cell proliferation and invasion by activating EZH2 in hepatocellular carcinoma. Biomedicine & Pharmacotherapy.

[71] Eisenach PA, Pedro Corrêa DS, Gillian M, Christian R (2012). Membrane type 1 matrix metalloproteinase (MT1-MMP) ubiquitination at Lys581 increases cellular invasion through type I collagen. Journal of Biological Chemistry.

[72] Pakravan N (2013). Tumorigenesis: Cell Defense Against Hypoxia?. Oncology Reviews.

