# Supplementary material for: Expression of MMP-14 and prognosis in digestive system carcinoma: a meta-analysis and databases validation
Source: J Cancer. 2020 Jan 1;11(5):1141–50. doi: 10.7150/jca.36469 (PMC6959085; doi:10.7150/jca.36469)
Supplement: Supplementary file 1 — Supplementary figures and tables. [file jcav11p1141s1.pdf]

Supplementary Table 1 The searching lists of search strategy.

No.	Keywords for database searching (Boolean operators)
1	“Carcinoma” [Topic] OR “Cancer” [Topic] OR “Tumor” [Topic] AND “Matrix metalloproteinases”AND “Prognosis” [Topic]
2	“Carcinoma” [Topic] OR “Cancer” [Topic] OR “Tumor” [Topic] AND “MT1-MMP”AND “Prognosis” [Topic]
3	“Carcinoma” [Topic] OR “Cancer” [Topic] OR “Tumor” [Topic] AND “MMP-14 or MT1-MMP”AND “Prognosis or Outcome or Survival” [Topic]
4	“Carcinoma” [Topic] OR “Cancer” [Topic] OR “Tumor” [Topic] AND “Matrix metalloproteinases or MMP-14 or MT1-MMP”AND “Prognosis” [Topic]
5	“'Carcinoma' or 'Cancer' or 'Tumor'” [Topic] AND “Follow-up studies” [Topic] AND “ Matrix metalloproteinases or MMP-14 or MT1-MMP” [Topic]
6	“MMP-14 or MT1-MMP” [Topic] AND “Hazard Ratio” [Topic] AND “' Matrix metalloproteinases or Carcinoma' or 'Cancer' or 'Tumor'” [Topic]

Supplementary Table 2 Quality assessment of included studies based on the Newcastle–Ottawa Scale for assessing the quality of cohort studies.

Study	Selection (score)				Comparability (score)	Exposure (score)			
	Representativenes Of the exposed cohort	Selection of the non-exposed cohort	Ascertainment of exposure	Outcome of interest was not present at start of study	Based on the design or analysis [a]	Assessment of outcome	Follow-up long enough for outcomes to occur	Adequacy of follow-up of cohorts	Total Score[b]
Cui et al [42]	1	1	1	1	2	1	1	1	**9**
Xu et al [43]	1	1	1	1	0	1	1	0	**6**
Bi et al [44]	1	1	1	1	2	1	1	0	**8**
Wang et al [45]	1	1	1	1	1	1	1	0	**7**
Zhang et al [46]	1	1	1	1	1	1	1	1	**8**
Zheng et al [47]	1	1	1	1	2	1	0	0	**7**
Naseh et al [48]	1	0	1	1	0	1	1	0	**5**
Zheng et al [49]	1	1	1	1	1	1	1	0	**7**
Dong et al [50]	1	1	1	1	1	1	1	0	**7**
Liu et al [51]	1	1	1	1	1	1	1	0	**7**
Akanuma et al [52]	1	0	1	1	0	1	1	0	**5**
Bao et al [53]	1	1	1	1	1	1	1	0	**7**
Liu et al [54]	1	1	1	1	1	1	1	0	**7**
He et al [55]	1	1	1	1	2	1	1	1	**9**
Peng et al [56]	1	0	1	1	0	1	1	1	**6**
Sun et al [57]	1	0	1	1	0	1	1	1	**6**
Yang et al [58]	1	0	1	1	0	1	1	1	**6**
Wu et al [59]	1	1	1	1	1	1	1	0	**7**
Huang et al [60]	1	1	1	1	1	1	1	1	**8**
Vicente et al[61]	1	1	1	1	2	1	1	1	**9**

[a] When there was no statistical significance in the response rate between case and control groups by using a chi-squared test ($P > 0.05$), one point was awarded.

[b]Total score was calculated by adding up the points awarded in each item.

Supplementary Table 3 Publication bias of MMP-14 for Begg's test and Egger's test.

Comparisons	Begg's test		Egger's test		
	z	*p*	*t*	*p*	95% CI
OS	2.49	0.14	1.93	0.107	-0.616-3.137
DFS/PFS	0.52	0.602	0.39	0.765	-59.45-58.43

Supplementary Table 4 The gene expression profile across all tumor samples and paired normal tissues (Mean).

No.	TCGA	Detail	Normal	Tumor
1	ACC	Adrenocortical carcinoma	27.02	19.18
2	BLCA	Bladder Urothelial Carcinoma	80.12	128.72
3	BRCA	Breast invasive carcinoma	86.62	141.88
4	CESC	Cervical squamous cell carcinoma and endocervical adenocarcinoma	154.55	118.74
5	CHOL	Cholangio carcinoma	6.35	125.06
6	COAD	Colon adenocarcinoma	59.49	85.69
7	DLBC	Lymphoid Neoplasm Diffuse Large B-cell Lymphoma	0.62	54.12
8	ESCA	Esophageal carcinoma	75.8	111.16
9	GBM	Glioblastoma multiforme	1.78	74.86
10	HNSC	Head and Neck squamous cell carcinoma	52.69	177.99
11	KICH	Kidney Chromophobe	24.64	15
12	KIRC	Kidney renal clear cell carcinoma	27.92	80.87
13	KIRP	Kidney renal papillary cell carcinoma	26.95	86.66
14	LAML	Acute Myeloid Leukemia	3.02	2.13
15	LGG	Brain Lower Grade Glioma	1.78	16.46
16	LIHC	Liver hepatocellular carcinoma	5	15
17	LUAD	Lung adenocarcinoma	88.54	84.85
18	LUSC	Lung squamous cell carcinoma	92.45	131.34
19	OV	Ovarian serous cystadenocarcinoma	84.07	52.67
20	PAAD	Pancreatic adenocarcinoma	13.27	226.94
21	PCPG	Pheochromocytoma and Paraganglioma	20.64	13.06
22	PRAD	Prostate adenocarcinoma	57.23	24.19
23	READ	Rectum adenocarcinoma	62.78	78.26
24	SARC	Sarcoma	48.02	416.58
25	SKCM	Skin Cutaneous Melanoma	102.3	266.06
26	STAD	Stomach adenocarcinoma	35.08	100.4
27	TGCT	Testicular Germ Cell Tumors	13	49.46
28	THCA	Thyroid carcinoma	54.73	44.31
29	THYM	Thymoma	0.62	39.55
30	UCEC	Uterine Corpus Endometrial Carcinoma	150.15	77
31	UCS	Uterine Carcinosarcoma	154.75	125.43

Legend for supplementary material

Supplementary Figure 1 Sensitivity analysis for OS of MMP-14 expression.

Supplementary Figure 2

A, Begg's funnel plot of publication bias on the relationship between MMP-14 expression and OS.

B, Egger's funnel plot of publication bias on the relationship between MMP-14 expression and OS.

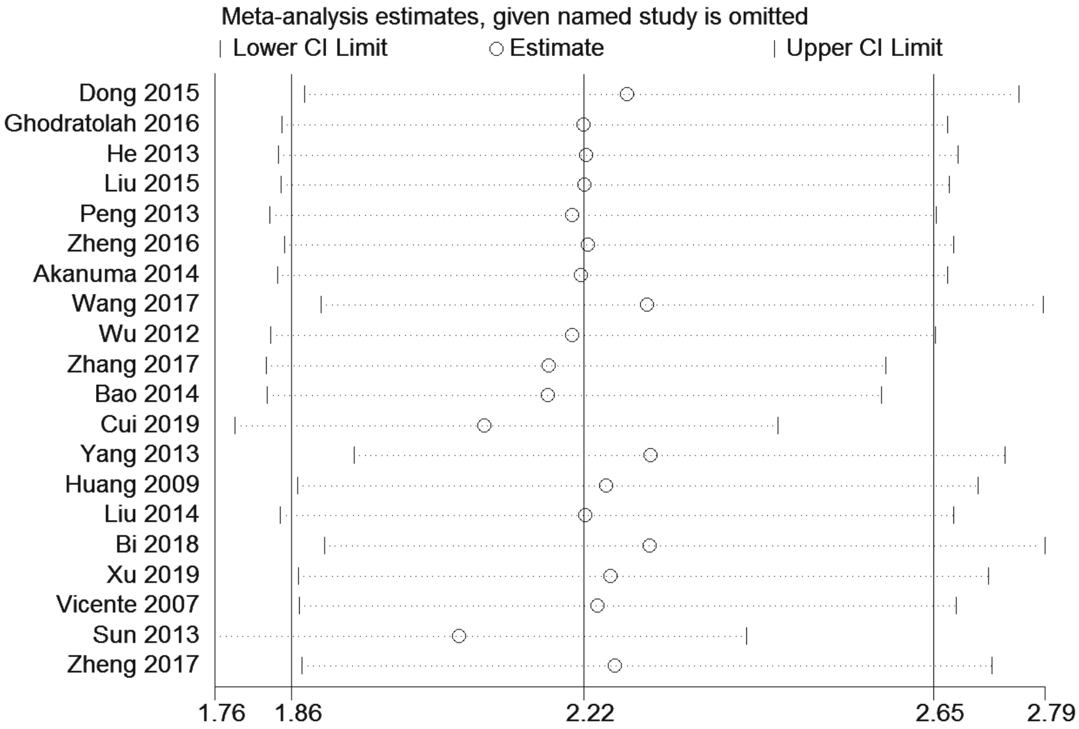

Supplementary Figure 1 Sensitivity analysis for OS of MMP-14 expression.

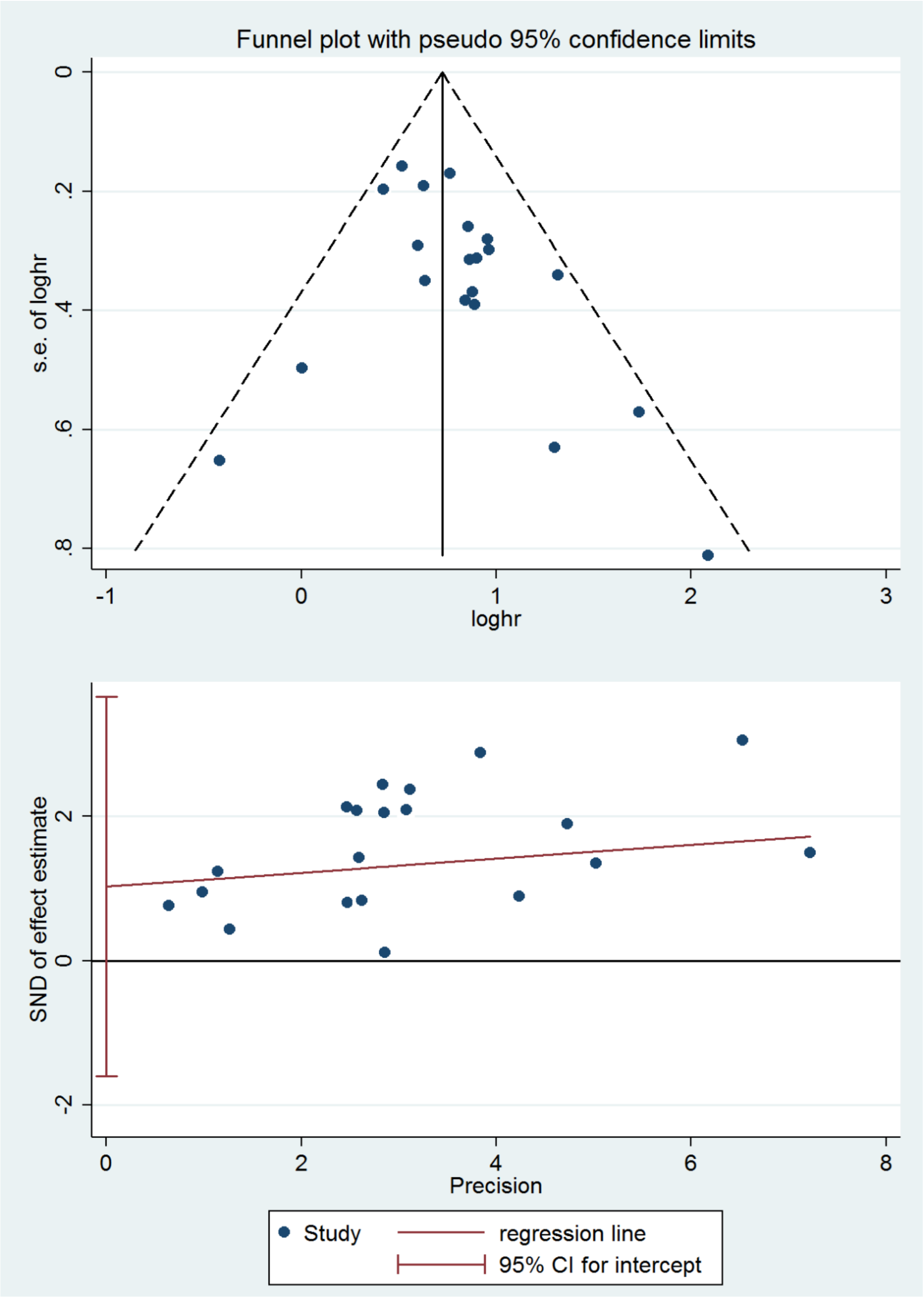

Supplementary Figure 2

A, Begg's funnel plot of publication bias on the relationship between MMP-14 expression and OS.

B, Egger's funnel plot of publication bias on the relationship between MMP-14 expression and OS.
